# News from South Africa

**Published:** 1920-08-28

**Authors:** 


### NEWS FROM SOUTH AFRICA.

### Free State National Hospital.

Increased hospital provision, or a new hospital, is urgently required for Bloemfontein, the Free State capital. Under the South African Act of Union hospitals were placed under the control of the four Provincial Councils. The Free State Provincial Council—a Hertzog Nationalist body—has during the last ten years, writes our correspondent, refused to do its duty in this respect, with the result that to-day the National Hospital at Bloemfontein is totally inadequate. The present accommodation is so inadequate that at least £110,000 is needed for the necessary additions. An effort is now being made to obtain funds by public subscription, and members of the board are leading the way. It is suggested that a new National Hospital—or additions to the present one—might be erected as a Free State War Memorial.

### Natal's Woman Medical Officer.

Dr. Mary Baird, M.B., Ch.B. (Edin.), who has been engaged from England as assistant medical officer of the Natal Education Department, has assumed duty at Pietermaritzburg. Dr. Baird has held several important hospital appointments in England. She was at one time resident medical officer of the Children's Hospital, Norwich, and house surgeon of the Children's Hospital, Derby. She was the senior house surgeon at the West Kent General Hospital, and during the late war served with the R.A.M.C. in Egypt. Dr. Baird went out to Natal direct from Torquay, where she was assistant medical officer of health and school medical officer.

